# Arthroscopic-assisted reduction and fixation of calcaneal fractures using sustentaculum tali and medial wall screws

**DOI:** 10.3389/fsurg.2025.1695657

**Published:** 2025-11-27

**Authors:** Zeyin Tang, Shuo Zhang, Nan Zhu, Chaoyue Huai, Xinru Wang, Junfeng Zhan, Juehua Jing

**Affiliations:** Department of Orthopedics, Institute of Orthopedics, Research Center for Translational Medicine, The Second Affiliated Hospital of Anhui Medical University, Hefei, China

**Keywords:** calcaneal fractures, arthroscopy, minimally invasive internal fixation, sustentaculum tali screw, medial buttress screw, Böhler angle

## Abstract

**Objective:**

This study retrospectively analyzed the clinical efficacy and safety of arthroscopy-assisted reduction and internal fixation in the treatment of displaced intra-articular fractures of the calcaneus (Sanders type II and III).

**Methods:**

Conducted at The Second Affiliated Hospital of Anhui Medical University, this retrospective study analyzed 60 consecutive patients with displaced intra-articular calcaneal fractures (DIACFs) admitted between January 2021 and February 2023. All patients underwent arthroscopically-assisted minimally invasive reduction and fixation with sustentaculum tali screws plus medial wall support screw. Outcomes were tracked preoperatively, postoperatively, and at final follow-up, encompassing radiographic metrics (Böhler angle, Gissane angle, calcaneal width, calcaneal height) and clinical scores (AOFAS Ankle-Hindfoot Scale, VAS). Pre-to-postoperative changes were analyzed using paired *t*-tests (normal distribution) or Wilcoxon signed-rank tests (non-normal distribution), with *P* < 0.05 considered significant.

**Results:**

All 60 patients completed a minimum 12-month postoperative follow-up. Fracture union was achieved in all cases with no loss to follow-up. Postoperative CT and x-ray imaging confirmed: satisfactory calcaneal morphological restoration, maintained reduction without significant loss between postoperative and final measurements (*P* > 0.05). At the end of the postoperative follow-up, The major complication rate was 0/60 cases (95% upper limit ≈ 4.9%); AOFAS score was 88.2 ± 3.9, with a 95% CI of 87.2–89.2; VAS score was 0.23 ± 0.57, with a 95% CI of 0.08–0.38.

**Conclusion:**

Arthroscopically assisted reduction and internal fixation using calcanar screws and medial wall support screws is a feasible and effective surgical strategy for the treatment of Sanders type II and type III intra-articular calcaneal fractures.

## Introduction

The calcaneus, being the largest tarsal bone, is the most frequent site for tarsal fractures. These fractures commonly arise from axial loading of the tibia or direct impact, such as from falls or traffic accidents, which can cause calcaneal collapse and comminution of the articular surface. Approximately 60% to 75% of calcaneal fractures are displaced intra-articular fractures, with high-energy, comminuted Sanders type II and III fractures being particularly prevalent (1). Inadequate reduction may lead to severe deformity of the calcaneal shape and articular surface, potentially resulting in post-traumatic subtalar arthritis and foot dysfunction.

Surgical intervention serves as the primary treatment for DIACFs aiming to reconstruct the calcaneal structure and restore articular surface morphology through reduction and fixation. The classic extended lateral “L-shaped” incision offers excellent exposure but necessitates extensive soft tissue dissection, with reported complication rates—including wound dehiscence, skin necrosis, and neurovascular injury—ranging from 11.7% to 35% (2). To mitigate trauma and reduce complications, various minimally invasive techniques have been developed. These include the sinus tarsi approach, percutaneous reduction combined with internal fixation, and reduction assisted by percutaneous bone levers or distractors (3).

The utilization of arthroscopic assistance in calcaneal fracture reduction and fixation has witnessed growing interest (4). Subtalar arthroscopy offers direct visualization of the posterior facet and lateral wall. Via minimally invasive portals, this technique permits precise disimpaction and reduction of the posterior facet utilizing elevators, evacuation of bone fragments and hematoma, and enhanced joint debridement. Intraoperative dynamic assessment of articular reduction enables timely adjustments to the fixation strategy, aiding in the restoration of normal Böhler and Gissane angles, thereby improving postoperative function. Furthermore, the sustentaculum tali fragment, commonly encountered in calcaneal fractures, is widely recognized for its dense bone structure and ligamentous stability, acting as a biomechanical strut for the posteromedial fracture block (5, 6). Placement of a sustentaculum tali screw can stabilize both the sustentaculum fragment and the posterior facet while elevating the articular surface. Additional studies propose the use of a medial wall buttress screw (inserted from the lateral calcaneus to buttress the medial wall) to further reinforce medial support, maintaining fracture reduction and preventing collapse. However, the clinical application of this technique remains limited.

## Study aim

Since January 2021, the Foot and Ankle Surgery Team at the Second Affiliated Hospital of Anhui Medical University has adopted arthroscopically assisted fixation utilizing sustentaculum tali screws and medial wall buttress screws to treat DIACFs (Sanders types II and III). This study retrospectively analyzes the clinical and radiographic outcomes of this cohort. Our objectives are to evaluate the clinical efficacy of this technique, including fracture reduction quality, fixation maintenance, functional recovery, and complication rates. Furthermore, by comparing our results with those reported in the literature, we aim to discuss the potential advantages and limitations of this approach relative to conventional surgical methods.

## Materials and methods

Study Design: We conducted a single-center retrospective case series study (adhering to STROBE guidelines) that included patients with Sanders II and III intra-articular calcaneal fractures who were consecutively admitted from January 2021 to February 2023. This study did not establish a randomized or parallel control group, and therefore only reports the correlation and feasibility evidence of short-term outcomes without making causal inferences. Key inclusion criteria comprised: (1) skeletal maturity (age ≥18 years); (2) acute fracture (<3 weeks from injury); (3) unilateral isolated injury (no concomitant ipsilateral ankle fractures or severe soft tissue compromise); (4) medically fit for surgery; (5) first presentation for fracture management. Exclusion criteria included open calcaneal fractures, old or malunited fractures, pathological fractures, multiple injuries requiring priority treatment of other lower limb fractures, and severe systemic diseases that contraindicated surgical intervention.

## Surgical technique

All procedures were performed by the same specialized foot and ankle surgical team. General anesthesia was utilized, followed by patient positioning in the lateral decubitus position with the contralateral side down. A tourniquet was applied at the thigh's root, set to 260 mmHg. Anterolateral and middle lateral arthroscopic portals were established to access the subtalar joint. Under arthroscopic visualization, a calcaneus fracture was observed, characterized by fragmentation and collapse of the lateral wall, with blood clots and synovium filling the joint cavity, thereby impairing its function. The synovium, fragmented bone fragments, blood clots, and inflammatory tissues were meticulously removed, subsequently allowing exposure of the posterior articular surface. Free fragmented bones were then excised, followed by thorough irrigation. Under arthroscopic guidance, precise repositioning of articular surface fracture fragments was conducted while elevating the calcaneal tuberosity approximately 2.0 cm to its anatomical position using temporary fixation with Kirschner wires. The anterior-lateral and lateral wall fracture fragments were under arthroscopic visualization before being temporarily fixed in place with Kirschner wires. C-arm fluoroscopy confirmed satisfactory reduction.

Percutaneous placement of sustentaculum tali screw(trans-calcaneal) and medial wall screw(inserted from the lateral calcaneus toward the medial wall) was executed for secure fixation. The positioning of these screws was verified as appropriate under both arthroscopic observation and C-arm imaging; notably, no screws penetrated into the joint cavity.

The surgical area underwent thorough rinsing to ensure hemostasis prior to suturing of incisions.

### Postoperative management and rehabilitation

Following surgery, the affected foot was immobilized in an elevated position, and antibiotics were administered prophylactically for 24 h to avert infection. No patients were immobilized using plaster casts. The rehabilitation protocol included instructing patients to initiate ankle pump exercises on the first postoperative day, to routinely change dressings from the third to the fifth postoperative day, and to undergo a follow-up radiographic assessment. Patients were discharged once the incision site exhibited no signs of redness, swelling, or exudation. Two weeks postoperatively, the patient's sutures were removed, and guidance was provided to gradually bear weight on the affected limb, progressed from partial to full weight-bearing over 8–12 weeks.

### Follow-up and outcome measures

All patients adhered to scheduled outpatient follow-up visits at specified intervals: 1, 3, 6, 12 months postoperatively and the final follow-up (which extended beyond 1 year for certain patients). The follow-up assessments encompassed clinical examination, radiographic evaluation, and functional assessment.

### Radiographic evaluation

Standard lateral and axial calcaneal radiographs were obtained at each follow-up visit. On these radiographs, we measured the Böhler angle (tuberosity-cuboid-articular surface intersection), Gissane angle (lateral process-dome-talar articulation), calcaneal height (distance between the tuberosity apex and cuboid articular surface), and calcaneal width (distance at the widest point of the calcaneus). Serial changes in these parameters were compared to assess the quality and maintenance of reduction. At final follow-up, a subset of patients underwent computed tomography (CT) to further evaluate subtalar joint alignment, assess for signs of post-traumatic arthritis (joint space narrowing, subchondral sclerosis, osteophyte formation), and confirm screw position (ensuring absence of intra-articular penetration, loosening, or breakage).

### Functional and pain assessment

Ankle-hindfoot functional outcomes were evaluated using the validated American Orthopaedic Foot and Ankle Society (AOFAS) Ankle-Hindfoot Scale. This 100-point scoring system quantifies three domains: Pain (40 points), Function (50 points), and Alignment (10 points). Outcomes were stratified into four categories according to final scores: excellent (≥90), good (75–89), fair (50–74), and poor (<50). The proportion of patients achieving ≥75 points, designated as the *good rate*, was subsequently derived. Pain intensity was measured with the Visual Analog Scale (VAS), where scores range from 0 (no pain) to 10 (maximal imaginable pain). Assessments documented both resting pain and pain during weight-bearing activities. Range of motion (ROM) was clinically measured to evaluate functional recovery, including active ankle dorsiflexion and plantarflexion, as well as subtalar joint inversion and eversion.

### Statistical analysis

Statistical analyses were performed using SPSS (Version 26.0). Using ΔBöhler, ΔGissane, and Δ calcaneal height or width as the primary outcomes, the mean difference and its 95% confidence interval (CI) were reported along with the paired effect size (Cohen's d). Normality was assessed using the Shapiro–Wilk test and Q–Q plots, and paired *t*-tests or Wilcoxon signed-rank tests were employed. Functional outcomes (AOFAS, VAS) were treated similarly and reported as mean ± 95% CI (AOFAS: 88.2 ± 3.9, 95% CI: 87.2–89.2; VAS: 0.23 ± 0.57, 95% CI: 0.08–0.38). Complication events were rare, and precise estimation was performed using the Clopper–Pearson interval (e.g., major complications 0/60, 95% upper limit ≈ 4.9%), without performing multivariate regression. All tests were two-tailed, and the significance threshold was set at 0.05.

## Results

### Patient characteristics and complications

All 60 patients (demographics summarized in Table 1) underwent successful surgery. Fracture union was achieved in all cases during the follow-up period, with no instances of delayed union or nonunion. The average wound heals in one stage, and the average operation time is 92.43 ± 16.57 min. All fracture patients had healed by the end of follow-up, All surgical incisions healed primarily without deep infection or local necrosis. Intraoperatively and during follow-up, no screw loosening or implant failure was observed. There were no reports of disabling neurovascular injuries, nor were there cases of delayed collapse under weight-bearing. During the follow-up period, 3 patients (Sanders III) experienced slight limitation in ankle mobility, and 1 patient reported significant early pain (with large blisters appearing on the soft tissue before surgery). However, at the final follow-up, all patients reported significant subjective pain relief and expressed high satisfaction with the results.

**Table 1:** Baseline patient characteristics.

Parameter	Value (*n* = 60)
Number of cases (*n*)	60
Age (years), mean ± SD	46.43 ± 9.74
Sex, Male/Female (*n*)	58/2
Injured side, Left/Right (*n*)	34/26
Mechanism of injury, Fall/MVA (*n*)	56/4
Hospital stay (days), mean ± SD	11.00 ± 2.86
Operative time (min), mean ± SD	92.43 ± 16.57

#### Imaging reduction quality an maintenance

Prior to the procedure, the Bohler Angle and Gissane Angle of all patients were significantly abnormal, indicating calcaneal collapse and deformation. Following arthroscopic-assisted reduction and double-screw fixation, x-ray measurements revealed that all indicators had significantly improved (refer to Table 2).

**Table 2:** Radiographic parameters preoperatively, postoperatively, and at final follow-up (mean ± SD).

Parameter	Preoperative mean ± SD	Immediate postoperative mean ± SD	Final follow-up mean ± SD	Δ Post–Pre	Δ Final–Post	95% CI of difference	Effect size dz	*P* value
Böhler angle (°)	7.10 ± 6.40°	30.73 ± 4.15°	30.53 ± 4.11°	23.63	−0.20	22.47–24.80	5.25	<0.001
Gissane angle (°)	106.30 ± 9.76°	125.77 ± 4.79°	125.37 ± 4.53°	19.47	−0.40	17.74–21.20	2.91	<0.001
Calcaneal height (mm)	41.08 ± 2.79	36.31 ± 2.56	36.45 ± 2.56	3.51	0.01	3.18–3.84	2.75	<0.001
Calcaneal width (mm)	39.03 ± 2.36	42.54 ± 2.36	42.55 ± 2.36	−4.77	0.14	−5.16–−4.37	−3.11	<0.001

Measurements labeled “Postoperative” refer to values obtained within 1 week post-surgery on follow-up radiographs. “Final Follow-up” measurements were obtained at the terminal assessment, conducted between 12 and 15 months postoperatively. An increase in the Böhler angle and calcaneal height indicates improved reduction. An increase in the Gissane angle indicates successful restoration of the normal posterior facet curvature. A decrease in calcaneal width indicates successful narrowing of the calcaneus towards its normal anatomy. *P*-values were derived from paired *t*-tests.

**In terms of functional scores** (refer to Table 3): the average AOFAS score at the last follow-up was (88.2 ± 3.9) points, and the VAS score was (0.23 ± 0.57) points (0–3 points). Over 90% of patients achieved a good or better outcome (AOFAS ≥ 85 points). Generally, the average fracture healing time was approximately 10 to 12 weeks, and all patients in the group underwent timely weight-bearing and functional exercises. All patients reported a high postoperative satisfaction rate, and none required a second operation. Short-term follow-up results indicated good functional recovery. No significant long-term complications were observed, although long-term monitoring is still necessary.

**Table 3:** Function scoring.

Score	Mean ± SD
AOFAS	88.20 ± 3.86
VAS	0.23 ± 0.57
Ankle dorsiflexion	8.23 ± 3.30
Ankle plantar flexion	22.23 ± 5.41

VAS < 1.0 indicates minimal clinical pain (IASP criteria).

#### Complications

This series did not report any major perioperative complications, such as significant neurovascular injury, compartment syndrome, deep infection, or implant failure. At final follow-up, radiographic evaluation revealed no evidence of post-traumatic subtalar arthrosis in any patient. All screws remained stable, showing no signs of loosening or migration, and there was no need for routine implant removal. No patient required secondary surgeries, such as subtalar arthrodesis, due to persistent pain. Although the patient expressed positive results during the recent follow-up, the follow-up period is relatively short, and it cannot be ignored whether problems such as arthritis may occur in the later stage. However, overall, patient outcomes were satisfactory, with a notably lower complication rate compared to those typically reported for conventional open techniques in the literature.

The findings of this study demonstrate that arthroscopically assisted reduction and fixation using sustentaculum tali screws with medial wall buttress screws for DIACFs (Sanders types II and III): preserves soft tissues through minimally invasive approaches, enables direct visualization of articular surface reduction, achieves precise anatomical restoration of calcaneal morphology and joint alignment. This technique represents a viable and effective alternative for the surgical management of these complex injuries.

## Discussion

### Soft tissue preservation: a key advantage of minimally invasive calcaneal fracture surgery

While the conventional extended lateral L-shaped approach for open reduction and internal fixation (ORIF) provides optimal visualization and remains the reference standard for achieving anatomical reduction, it carries a significant risk of surgical site morbidity, substantially impeding rehabilitation. Complication rates as high as 20%–25% are documented for open techniques (7), including wound dehiscence and deep infection, which may necessitate prolonged management or reconstructive procedures.

Conversely, minimally invasive strategies—exemplified by the sinus tarsi approach combined with percutaneous screw fixation—have gained increasing adoption. This shift is driven by their demonstrated efficacy in significantly reducing soft tissue complications (8, 9). Comparative studies and meta-analyses corroborate this benefit, confirming that internal fixation via the sinus tarsi approach achieves reduction quality comparable to the traditional extended lateral method. For instance, Rammelt et al. (10) reported a substantially lower wound complication rate in their minimally invasive cohort vs. the open surgery group (*P* < 0.001), with no significant differences in postoperative radiographic parameters (Böhler angle, Gissane angle) or functional recovery (AOFAS score) at follow-up. Collectively, this evidence indicates that minimally invasive techniques maintain reduction precision while significantly enhancing perioperative safety, supporting their expanded clinical implementation. At our center, the fixation protocol incorporates sustentaculum tali screws and medial wall buttress screws. Soft tissue trauma is inherently minimized by the limited incision size required, and extensive dissection of the calcaneal region is circumvented. Consequently, surgical delay could be reduced, even in cases where significant preoperative swelling or fracture blisters were present. Consistent with existing literature, no major wound complications were recorded in our case series, which exclusively employed minimally invasive techniques. Furthermore, both hospitalization duration and overall recovery time were significantly abbreviated relative to historical outcomes from open surgical procedures.

### Advantages of arthroscopic assistance

Building upon minimally invasive principles, the integration of arthroscopy offers a transformative perspective in calcaneal fracture management. Arthroscopic visualization enables direct assessment of subtalar joint surface reduction—a critical advantage unattainable through fluoroscopy alone. High-energy intra-articular calcaneal fractures frequently present with severe post-traumatic swelling and poor soft tissue conditions (e.g., tension blisters), which often delay surgery. Our technique—arthroscopically assisted fixation using sustentaculum tali screws with medial wall buttress screws—demonstrates a distinct reduction in preoperative waiting time compared to other minimally invasive approaches. The reduction tool we use is only the Kerner's needle, which does not have such high requirements for surgical instruments. Although the tool is simple to use, it does test the surgeon's imagination of the morphological structure of the calcaneus. However, this is more friendly to some surgeons with less strength. The arthroscope provides unparalleled visualization of comminuted posterior facet fractures, allowing surgeons to identify residual step-offs or gaps as small as 1–2 mm. This facilitates precise intraoperative adjustments until articular continuity is restored. The direct visualization inherent to arthroscopy: Enhances anatomical reduction rates of articular surfaces, Prevents oversight of intra-articular fragments, Optimizes reconstruction of calcaneal morphology, Maintains reduction stability throughout healing. This minimally invasive approach demonstrates a favorable complication profile and satisfactory short-term outcomes, supporting its clinical utility for Sanders type II–III calcaneal fractures. Despite the potential advantages of arthroscopy-assisted surgery in terms of soft tissue complications, it has a steep learning curve, potentially longer operation times in the early stages, and increased costs. Differences in surgeon experience and resources across different centers may affect reproducibility and generalizability. Similarly, the severity of a patient's fracture increases the difficulty of the required debridement and reduction. The cases we reviewed so far show that Type II and Type III fractures have similar improvements in short-term function and imaging (referring to the last follow-up), with notable differences being slightly longer operation times and fluoroscopy times for Type III fractures. During the follow-up period, patients with Type III fractures had to wait longer for full weight-bearing compared to those with Type II fractures. Therefore, we did not conduct a detailed subtype analysis. In the future, once the surgical method is widely adopted, we plan to conduct a multicenter retrospective cohort controlled study with a larger number of cases and a more balanced distribution of male and female patients, as well as different fracture subtypes.

### Significance of dual-column screw fixation

**Figure 1:**
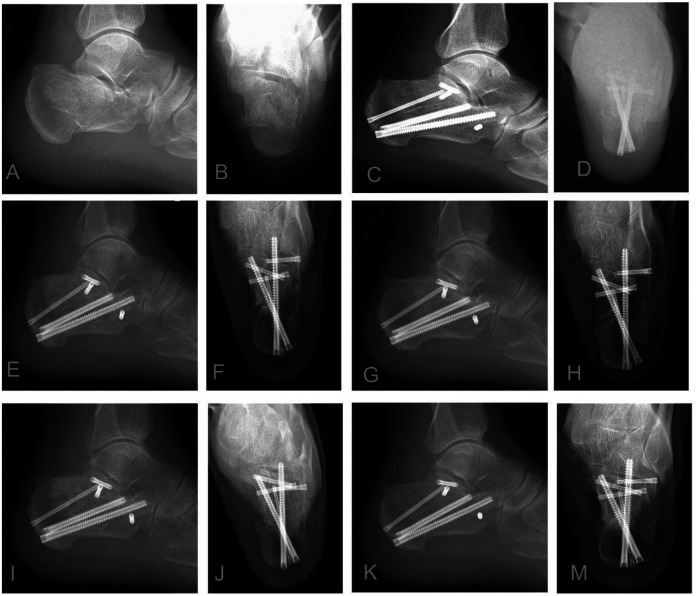
Illustrative case: A 55-year-old male sustained a left sanders type IIB displaced intra-articular calcaneal fracture. (**A,B**) Preoperative lateral and axial radiographs: Reveal fracture comminution, loss of calcaneal height, increased width, and varus deformity. (**C,D**) Immediate postoperative radiographs: Demonstrate restoration of calcaneal height and width, anatomical reduction of the posterior facet, correction of varus deformity, and optimal implant positioning (sustentaculum tali screws and medial wall plating). (**E–M**) Serial postoperative radiographs (1, 3, 6, 12 months): Document maintained fracture reduction, stable implant position without evidence of loosening or migration, and progressive fracture union without collapse. Arthroscopic confirmation noted a posterior facet step-off <1 mm.

Our strategy, employing a dual-screw construct (sustentaculum tali screw combined with medial wall buttress screw fixation), aims to augment stability following arthroscopically assisted reduction. Maintaining this fixation stability is critical for preserving reduction throughout fracture union. In this series, the construct successfully maintained the restored calcaneal morphology: Radiographic parameters (Böhler angle, Gissane angle, calcaneal height, width) demonstrated no significant loss compared to immediate postoperative values at a mean follow-up of 6 months. There was no evidence of posterior facet re-collapse or calcaneal deformity recurrence. This indicates that the two screws provided sufficient biomechanical strength to maintain fracture alignment during the pre-weightbearing healing phase. This finding aligns with the literature. A randomized controlled trial by Rammelt et al. (11) demonstrated that adding a sustentaculum tali (ST) screw to conventional plate fixation significantly improved postoperative AOFAS scores, reduced pain, and resulted in less Böhler angle loss at 1-year follow-up. Similarly, Sun et al. (12) demonstrated that adding a sustentaculum tali (ST) screw to conventional plate fixation significantly improved postoperative AOFAS scores. According to the results of our treatment group's follow-up visits, although we instructed patients to start bearing weight at 8 weeks, according to our follow-up records, some patients actually began to walk with full weight-bearing earlier than this time. Two patients were even walking with full weight-bearing at the 1-month postoperative follow-up, which exceeded our imagination. We consider that it is more likely that the medial wall screws played a decisive role by providing stronger support to the calcaneal articular surface. However, the current sample size is small, so we cannot rule out the possibility of accidental events. Although our study lacked a control group, our follow-up data similarly demonstrated minimal Böhler angle loss (Figure 1), indicating excellent maintenance of reduction. Compared to isolated lateral fixation, a trans-calcaneal screw engaging the medial wall provides direct buttressing of the crucial medial sustentacular fragment. This effectively compensates for the limited medial exposure inherent to the sinus tarsi approach. Our technique eliminates plate fixation, relying solely on two strategically placed large-diameter screws to achieve bipodal support across the medial and lateral columns. This theoretically provides comparable stability while avoiding plate-related complications such as soft tissue irritation and the frequent need for subsequent implant removal. Technical Consideration: The spatial orientation of the dual screws requires careful planning. Intraoperative fluoroscopy must confirm that: The screws do not interfere with each other, Neither screw penetrates the subtalar joint space, Both screws adequately engage the key fracture fragments. Suboptimal screw positioning should prompt intraoperative revision, as malpositioned screws may fail to provide adequate support and potentially lead to complications.

### Functional recovery and complication profile: evidence from comparative literature

Current research on arthroscopically assisted calcaneal fracture fixation remains limited to retrospective case series with modest sample sizes. Several comparative studies, however, have directly contrasted this technique with open approaches. Yang et al. (13) compared arthroscopic cannulated screw fixation (SJACF) and extended lateral approach plating (ELA) for Sanders type II–III fractures, demonstrating comparable radiographic outcomes: postoperative Böhler angle, Gissane angle, and calcaneal dimensions showed no significant differences between groups at all follow-up intervals (*P* > 0.05). Both groups showed significant improvement over preoperative values. Key Implications: With proper reduction technique, minimally invasive approaches achieve anatomical restoration comparable to open surgery. Perioperative Metrics: Hospital Stay: Significantly shorter in arthroscopy group Fluoroscopy Usage: Significantly reduced in arthroscopy group Operative Time: Slightly longer in arthroscopy group (reflecting technical complexity and learning curve) Clinical Key advantages of the minimally invasive approach include significantly lower postoperative pain scores, accelerated functional recovery, and a reduced incidence of complications—notably soft tissue issues such as wound dehiscence and infection—compared to conventional open techniques. Zeng et al. (14) evaluated arthroscopic bioabsorbable screw + K-wire fixation vs. modified sinus tarsi approach screw fixation: Both groups achieved excellent fracture union and significant improvement in pain or functional scores. Functional Superiority: Arthroscopy group demonstrated significantly higher AOFAS and Maryland Foot Scores at 3 and 12 months. Complication Contrast: Arthroscopy Group: 1 case of post-traumatic arthritis (2.5%). Control Group: 10% incidence of subtalar pain (primarily peroneal tendinitis). Consistent with Our Findings: arthroscopic assistance enables: more thorough articular debridement. Precise reduction of joint surfaces. Potential prevention of postoperative joint pain and tendon irritation. These findings align with recent studies (Stødle et al., Younger et al., Su et al.) (15–17) corroborate: Lower postoperative pain scores, accelerated recovery timelines, preservation of functional outcomes, robust safety profile for arthroscopic techniques.

#### Supporting studies and evolving trends

Beyond the core literature discussed, multiple studies have further validated the feasibility and expanding applicability of arthroscopically assisted calcaneal fracture management across radiographic outcomes, technical innovations, and patient selection: Restoration Pastides et al. (18), in a decade-long retrospective analysis, demonstrated that percutaneous and arthroscopically assisted techniques achieved superior Böhler angle reconstruction compared to conventional approaches (32.1° ± 3.8° vs. 28.5° ± 4.2°, *P* = 0.007). Woon et al. (19) introduced a novel percutaneous arthroscopic reduction technique minimizing soft tissue disruption while improving postoperative stability (wound complication rate: 2.1% at 2-year follow-up). Wong et al. (19) and Law et al. (20) developed hybrid fluoroscopic-arthroscopic navigation systems, enhancing visualization during percutaneous fixation and reducing complications in complex fracture patterns (malreduction rate: 3.4% vs. 12.7% in conventional groups). Gao et al. (21) pioneered intraoperative distraction devices to augment arthroscopic working space, facilitating intervention in severely comminuted intra-articular fractures (joint surface step-off <1 mm achieved in 93% of cases). Rammelt et al. (10, 11) systematically established the biomechanical rationale for arthroscopy in low-to-moderate complexity fractures, laying the foundation for modern minimally invasive protocols. Building on this rationale, clinical comparisons favor the sinus tarsi approach: a large RCT demonstrated earlier surgery, shorter operative times, faster union, and fewer wound complications and post-traumatic subtalar arthritis with STA, alongside better radiographic restoration and higher AOFAS scores Fadle et al. (22). The results of our short-term follow-up show that the complications of arthroscopic surgery are lower. However, due to the short follow-up period, we will extend the follow-up period in the future to compare with it. Dai et al. (23) extended this approach to high-risk populations (e.g., diabetics), confirming significantly lower complication rates vs. open techniques (infection: 0% vs. 18%, *P* < 0.01). Synthesis: These advancements collectively underscore the broad applicability and promising clinical trajectory of arthroscopically assisted techniques for DIACFs.

### Multidisciplinary management and differential diagnosis

In addition, the persistence and recurrence of symptoms such as chronic heel pain may be related to hidden factors beyond bone morphology, such as involvement of the lateral plantar branch caused by Baxter nerve compression. This issue has been emphasized as the “neglected culprit” in recent reviews, suggesting that neurological examination and rehabilitation strategies such as gait or insole assessment should be included in postoperative evaluations (24). Another example is the latest editorial on combined distal tibiofibular injuries, which emphasizes the integration of surgical, rehabilitation, and podiatric interventions to optimize prognosis, highlighting the multidisciplinary trend in ankle and foot disease management (25). These perspectives remind us that perioperative and follow-up management of intra-articular calcaneal fractures should not be limited to bone reconstruction alone, but should also incorporate pain neurogenic factors and a multidisciplinary rehabilitation approach. This helps us to pay more attention to whether direct or indirect nerve compression or injury occurs during the treatment of fracture patients, thereby alleviating postoperative pain and facilitating rehabilitation therapy. Arthroscopic reduction of intra-articular calcaneal fractures allows direct visualization of the fracture ends with minimal soft tissue sacrifice, providing surgeons with a clearer view for neurovascular protection.

### Study limitations

This single-center retrospective case series has several inherent limitations: (1) Retrospective without control: Only feasibility and associative evidence are provided; we provide indirect comparison with published studies but do not make causal inferences. Prospective controlled or randomized studies are needed in the future. (2) Small sample size and gender imbalance: Subgroup exploration is reported and limitations of extrapolation are acknowledged; in the future, multicenter studies will expand the sample size of women and elderly patients. (3) Short follow-up: Only reflects short-term safety and function; follow-up will be extended to ≥5 years to assess subtalar joint degeneration. (4) Non-blind radiographic review: A two-rater consistency protocol is adopted to reduce bias. (5) Single team: Results may be influenced by the learning curve, requiring cross-center validation.

#### Future research

Multi-center prospective controlled/randomized studies (including propensity score matching), expanding the sample size of women and elderly patients; follow-up for ≥5 years to assess subtalar joint degeneration and long-term function; incorporating cost-effectiveness analysis and learning curve evaluation.

## Conclusion

In this single-center retrospective case series, arthroscopically assisted treatment with talar calcaneal screw fixation combined with medial wall support was employed for Sanders II and III intra-articular calcaneal fractures. Short-term follow-up indicated good radiographic reconstruction and functional improvement, with no significant major complications observed. This approach promotes reliable fracture union, significantly improves postoperative function, and minimizes incision-related complications, aligning with contemporary minimally invasive principles. Based on systematic reviews and recent clinical evidence (13, 26–29) this technique is recognized as a safe, effective strategy adhering to modern minimally invasive standards. It demonstrates particular efficacy in restoring the calcaneus's physiological angles and morphology. However, due to limitations in study design, sample composition, and follow-up duration, we can only provide evidence of feasibility and short-term safety. Future multi-center, prospective controlled studies and longer-term follow-ups are warranted to further validate the robustness and generalizability of the treatment efficacy.

## Data Availability

The original contributions presented in the study are included in the article/Supplementary Material, further inquiries can be directed to the corresponding author/s.
